# Changes in liraglutide-induced body composition are related to modifications in plasma cardiac natriuretic peptides levels in obese type 2 diabetic patients

**DOI:** 10.1186/1475-2840-13-36

**Published:** 2014-02-05

**Authors:** Chun-Jun Li, Qian Yu, Pei Yu, Tie-Lian Yu, Qiu-Mei Zhang, Shan Lu, De-Min Yu

**Affiliations:** 1 Department of Endocrinology, 2011 Collaborative Innovation Center of Tianjin for Medical Epigenetics, Key Laboratory of Hormone and Development (Ministry of Health), Metabolic Disease Hospital & Tianjin Institute of Endocrinology, Tianjin Medical University, Tianjin 300070, China; 2 Department of Radiology, 2011 Collaborative Innovation Center of Tianjin for Medical Epigenetics, Key Laboratory of Hormone and Development (Ministry of Health), Metabolic Disease Hospital & Tianjin Institute of Endocrinology, Tianjin Medical University, Tianjin China; 3 Department of Radiology, General Hospital affiliated Tianjin Medical University, Tianjin China

**Keywords:** Liraglutide, Body composition, Weight loss, Cardiac natriuretic peptides

## Abstract

**Background and aims:**

Liraglutide treatment can improve glycemic control with a concomitant weight loss, but the underlying mechanism on weight loss is not completely understood. Cardiac natriuretic peptides (NPs) can resist body fat accumulation through increasing adipocytes lypolysis. In this study, we tested the hypothesis that liraglutide-induced weight loss was associated with increased plasma NPs concentrations.

**Methods:**

Thirty-one outpatients with type 2 diabetes (T2D) treated with metformin and other oral antidiabetic drugs except for thiazolidinediones (TZDs) were subcutaneously administered with liraglutide for 12 weeks. Body composition, abdominal visceral adipose tissue areas (VAT) and subcutaneous adipose tissue areas (SAT) were assessed at pre- and post-treatment by dual-energy X-ray absorptiometry (DXA) scanning and abdominal computerized tomography (CT). Plasma atrial natriuretic peptides (ANP) and B-type ventricular natriuretic peptides (BNP) concentrations were tested by commercial ELISA Kit quantitatively.

**Results:**

Following 12-week liraglutide treatment, body weight, waist circumference, total fat and lean mass, fat percentage, SAT and VAT areas were significantly reduced from baseline. Concurrently, plasma ANP and BNP levels were significantly increased following 12-week liraglutide treatment. There were significant correlations between the reductions in body compositions and the increases in both plasma ANP and BNP levels.

**Conclusions:**

There were significant correlations between increases in both plasma ANP and BNP levels and changes in liraglutide-induced body composition. Our data implied that increases in plasma NPs may add a novel dimension to explain how liraglutide induces weight loss.

## Introduction

The prevalence of obesity and diabetes has rapidly increased worldwide including Western and Asian countries, it is estimated that 80% of type 2 diabetic patients are obese [1]. In China, the combined prevalence of overweight and obesity has increased nearly 50% in a 10-y period [2]. In this sense, it is necessary to develop effective and efficient therapeutic strategy target for type 2 diabetes and obesity. Liraglutide, glucagon-like peptide-1 (GLP-1) analogue, is a member of the newest class of T2D therapies currently available, which improve hyperglycemia through increasing insulin secretion and reducing glucagon secretion [3], slowing gastric emptying, delaying carbohydrate absorption, and increasing satiety, leading to reduced caloric intake [4]. The Liraglutide Effect and Action in Diabetes (LEAD)-2 and LEAD-3 studies have demonstrated that liraglutide treatment produced sustained improvements in glycaemic control with a concomitant sustained weight loss and very low risk of hypoglycemia [5,6]. The DXA and CT assessments have shown that reductions in body weight with liraglutide primarily come from reductions in fat mass rather than lean tissue mass, furthermore, abdominal visceral fat tissues (VAT) reduced greater than subcutaneous fat tissues (SAT) [7]. Therefore liraglutide might be a promising new agent for the treatment of T2D and abdominal obesity linked to high risk of cardiovascular disease (CVD). In a pilot study, we recently reported that liraglutide treatment led to a mean reduction in body weight of 5.62 kg in Chinese obese T2D [8], much greater than reported in LEAD-2 and LEAD-3 studies [5,6], but the effect of liraglutide-induced weight loss on body composition has not been observed in Chinese T2D. In addition, the underlying mechanism on liraglutide-induced weight loss is not completely understood.

The mechanisms underlying the weight-loss observed with liraglutide are likely to be similar to those of native GLP-1, potentially by slowing of gastric emptying [9], increasing perception of satiety [10] and nausea [11], and/or changing the secretion of other gastrointestinal hormones [3]. A mean weight loss of up to 7.8 kg has also been demonstrated in non-diabetic obese subjects treated with liraglutide for 20 weeks and was apparently not only due to induction of nausea and less energy intake [12]. In vitro study, GLP-1 was certified to increase lipolysis in adipocytes in a dose-dependent manner [13]. In clinical study, Shalev and colleagues observed an increase in resting energy expenditure of 27% with peripheral GLP-1 administration in healthy men [14]. Recently, Minsuk Kim et al. demonstrated that cardiac GLP-1R activation promotes the secretion of atrial natriuretic peptide (ANP) [15]. The cardiac natriuretic peptides (NPs), ANP and BNP, are well known for key hormones in fluid and hemodynamic homeostasis. Increases in circulating NPs were also shown to be associated with increased postprandial fat oxidation, adipocytes lipolysis and resultant weight loss in both healthy and obese humans [16-18]. Furthermore, cross-sectional studies shown that obese individuals have lower natriuretic peptide concentrations than individuals of normal weight [19,20], and patients with metabolic syndrome often show reduced circulating concentration and biological efficacy of NPs [21], indicating that obese individuals have relatively natriuretic deficiency. Taken together, such studies led to an attractive hypothesis that low plasma NPs levels might be associated with obesity,and the capacity of promoting lipolysis makes it an attractive target for anti-obesity therapies [22].

The increase in glucagon-like peptide-1 (GLP-1) activity has emerged as a useful therapeutic tool of achieving and maintaining good gylcemic control with concomitant weight loss in T2D with obesity [5,6,8]. In this study, we investigated whether liraglutide-induced weight loss was associated with increased circulating NPs levels and provided new insights into its underlying mechanism of weight loss.

## Methods

### Study participants

This was a prospective, 12-week observational study of subjects with T2D and obesity who were administrated subcutaneous liraglutide as add-on therapy. Patients were recruited from three diabetes specialists in the out-patient setting of the Metabolic Disease Hospital of Tianjin Medical University between September 2012 and June 2013. The inclusion criteria were: 1) known T2D with; 2) obesity (BMI ≥ 28 kg/m^2^); 3) HbA1c 7.0 ~ 10%, and 4) at least 3 months treatment on a stable dose regime of maximal dose of metformin, or combined with either insulin, or any other oral anti-diabetes drugs except for TZDs. The key exclusion criteria were: 1) a history of coronary artery disease based upon a history of myocardial infarction, stable angina, congestive heart failure or unstable angina documented in physician notes or cardiac catheterization, 2) significant renal impairment (estimated creatinine clearance <60 ml/min or serum creatinine >150 μmol/l) or liver damage (serum alanine or aspartate aminotransferase three of more times the upper-normal range); 3) treatment within the last 3 months with pioglitazone, orlistat, any other drugs known to affect weight control, including glucocorticoids, or tamoxifen.

During the 12-week study period, 31 of 35 eligible subjects continued their usual diet and exercise regimens as well as any concomitant glucose-lowering medications. All patients were treated with the maximal metformin, 6 with alpha-glucosidase inhibitor, 9 with glinide, 10 with glimepiride and 6 with dipeptidyl peptidase-IV (DPP-IV) inhibitor. Addition liraglutide was initiated at 0.6 mg once daily, titrated to 1.2 mg once daily after one week. Insulin doses or oral anti-diabetes drugs were reduced 0 ~ 50% upon initiation of liraglutide based upon the prescribing diabetes specialists’ judgement. They could first decide to discontinue oral insulin secretagogues in the events of hypoglycemia or hypoglycemic symptoms occurred in the daytime; if hypoglycemia occurred in the nighttime, the investigators could consider decreasing insulin dose or discontinuing insulin treatment according to their clinical judgments. All subjects remained under the supervision of the diabetes specialist team throughout the study. The study protocol was approved by the Tianjin Medical University Ethics Committee Review Board and was conducted in accordance with the Declaration of Helsinki and Good Clinical Practice guidelines. All participants gave their written informed consent before beginning the study.

### Clinical examination

All of the blood draws were obtained in the fasted state before and after 12-week liraglutide treatment. For the determination of changes of plasma ANP and BNP, pre- and post-treatment samples from the same patient were paired. The blood samples were taken and immediately cooled and centrifuged at 4°C, then stored at -80°C until analysis.

HbA1c was analyzed using a high-performance liquid chromatography, ion-exchange chromatography assay (HLC-723G7, TOSOH, Japan). Serum concentrations of insulin and C-peptide were analyzed by enzyme linked immuno sorbent assay (ELISA) methods. Serum glucose, lipid profiles and liver biochemistry were determined by using the Hitachi 7070 automatic biochemical analyzer (Hitachi Ltd, Japan). Plasma ANP and BNP levels were quantified by commercial ELISA Kit (Biovendor, Mordrice, Czech Republic) performed according to the manufacturer’s instructions. Each sample was run in triplicates and the mean value was obtained by calculation using the standard curve method. Paired pre- and post-treatment samples were run on the same ELISA plate to minimize plate-related assay variation. The detection limits of the ELISA were 2.3 pg/ml for ANP plasma levels and 1.02 pg/ml for BNP levels with an intra-assay coefficient of variation (CV) of (7.8% ~ 9.9%) and interassay CV of (8.1% ~ 15%) for both ANP and BNP.

### Body composition

Body weight was measured at all visits without shoes and wearing only light clothing by Tanita scales analyzer (Tanita TCS-WB-3000, UK). Height was measured using a stadiometer to the nearest 0.5 cm. Waist circumference was taken at the midpoint between the anterior superior iliac spine and the lower edge of the rib cage. A single observer, who was not involved in the clinical care of the patients, made all of the above measurements. Lean tissue mass, fat mass, and total body weight were assessed before treatment by a low radiation DXA (GE Prodigy, WI USA) scan in all fasting subjects and again after the 12-week liraglutide intervention period. Abdominal visceral and subcutaneous adipose tissue areas measured by single-slice abdominal CT have been shown to have a strong direct linear correlation with visceral and abdominal subcutaneous tissue mass respectively [23], allowing for changes in these two tissue types to be compared. At baseline and after treatment, subjects were placed in the supine position and a truncal CT scan was performed using GE Discovery CT 750 HD scanner (GE Healthcare, Milwaukee, WI, USA) with slice thickness set at 5 mm. The images were analyzed using image analysis software (ImageJ, version 1.43q; National Institutes of Health, Bethesda, MD, USA) with an attenuation range of −50 to −250 Hounsfield units to quantify the subcutaneous, visceral, and total abdominal adipose tissue areas at the level of the intervertebral disc between lumbar vertebrae four and five (L4:L5). The results were expressed in centimeter square. The visceral/subcutaneous adipose tissue area ratio (VAT/SAT ratio) was calculated. All DXA and CT scans were performed by a single specialist using the same equipment and assessed by a single reader who was blinded to the study question, patient and follow-up time point.

### Statistical analysis

Normally distributed data are expressed as mean ± standard deviation and non-normally distributed data as median or as numbers and percentages. Non-normally distributed data were log transformed for use with parametric statistics. Paired t-tests were used to assess the differences in body composition, cardiac NPs levels and laboratory parameters between individuals at pre- and post-treatment (pre - post) and are presented as mean differences with 95% confidence intervals. Unpaired t-tests were used to compare the changes between different body composition intra-individuals after treatment and the difference between subgroups. Pearson correlation analysis was used to assess possible relationships between alterations of plasma cardiac NP levels and body composition and other laboratory data. The statistical analyses were performed using SPSS windows version 17.0, and p value < 0.05 was considered to be statistically significant.

## Results

### Characteristics of participants

31 of the 35 eligible subjects who were underwent baseline scanning completed the study. The 4 dropouts initiated treatment after baseline assessments, one declined follow-up, one changed hospital and two discontinued GLP-1 treatment due to gastrointestinal side effects. Table 1 shows baseline characteristics of 31 subjects (16 male, 15 female) at admission. The mean age was 48.5 ± 11.4 years, the mean glycated hemoglobin (HbA1c) was 8.2 ± 0.77%, and mean body mass index (BMI) was 31.7 ± 3.6 kg/m^2^. All patients were treated with the maximal tolerated dose of metformin before recruitment and they remained on the same mean dose throughout the study. The oral anti-diabetes agent histories of subjects were seen in Table 1. These parameters indicated that the enrolled subjects had severe abdominal obesity and poor glycemic control.

**Table 1:** Baseline characteristics

**Variable**	**Baseline (n = 31)**
Sex (Male/Female)	16/15
Age (years)	48.5 ± 11.4
Duration of diabetes (years)	6.9 ± 4.1
HbA_1c_ (%)	8.2 ± 0.77
Body weight (kg)	91.6 ± 12.4
BMI (kg/m^2^)	31.7 ± 3.6
Waist circumference (cm)	108.7 ± 8.8
Medications for Diabetes	
Metformin (n, %)	31 (100%)
Sulphonylurea (n, %)	10 (32.3%)
Alpha-glucosidase inhibitor (n, %)	6 (19.4%)
Glinide (n, %)	9 (29.0%)
DPP-IV inhibitor (n, %)	6 (19.4%)

Normally distributed data expressed as mean ± standard deviation and non-normally distributed data expressed as median or as numbers and percentages. HbA1c: glycosylated haemoglobin A1c. BMI: body mass index.

### Changes in body composition following 12-week liraglutide treatment

Body weight, waist circumference and BMI were significantly decreased from baseline to post-treatment with liraglutide in all subjects, the mean reductions in body weight, waist circumference and BMI were 5.03 kg, 3.00 cm and 1.74 kg/m^2^ respectively (Table 2). 61.3% (n = 19) of the subjects in the study lost more than 5% of body weight from baseline. There was no correlation between the pre-treatment body weight and the reduction in body weight (r = -0.27, p = 0.886).

**Table 2:** Changes in body composition and metabolic parameters following 12-week liraglutide treatment

**Variable**	**Pre-treatment (n = 31)**	**Post-treatment (n = 31)**	**Mean changes from baseline (95% CI)**	**p value**
Weight (kg)	91.6 ± 12.4	86.5 ± 12.5	-5.03 (-5.81, -3.80)	<0.001
BMI (kg/m^2^)	31.7 ± 3.6	29.9 ± 3.8	-1.74 (-2.02, -1.31)	<0.001
waist circumference (cm)	108.7 ± 8.8	105.7 ± 9.2	-3.00 (-3.89, -2.11)	<0.001
Total fat mass (kg)	33.77 ± 10.33	29.98 ±10.76	-3.79 (-4.53, -3.05)	<0.001
Total lean mass (kg)	54.88 ± 8.42	53.36 ± 8.34	-1.52 (-2.03, -1.10)	<0.001
Relative total body Fat (%)	37.89 ± 8.22	35.60 ± 9.01	-2.29 (-2.91, -1.66)	<0.001
Relative total body Lean (%)	62.06 ± 8.22	64.39 ± 9.01	2.34 (1.67, 3.01)	<0.001
Abdominal VAT areas (cm^2^)	277.80 ± 68.75	235.49 ± 65.62	-42.31 (-50.28, -34.33)	<0.001
Abdominal SAT areas (cm^2^)	212.65 ± 83.17	189.35 ± 83.37	-23.29 (-29.43, -17.16)	<0.001
VAT:SAT ratio	1.41 ± 0.62	1.34 ± 0.64	-0.06 (-0.11, -0.02)	0.009
HbA1c (%)	8.19 ± 0.77	7.13 ± 0.54	-1.06 (-1.25, -0.87)	<0.001
FBG (mmol/L)	8.63 ± 1.20	6.86 ± 0.73	-1.77 (-2.16, -1.39)	<0.001
P2BG (mmol/L)	13.05 ± 2.72	8.66 ± 0.91	-4.39 (-5.24, -3.54)	<0.001
sFCP (ng/mL)	2.23 ±1.17	3.51 ±1.80	1.27 (0.73, 1.81)	0.001
CPI	1.47 ±1.01	2.74 ±1.47	1.27 (0.58, 1.97)	<0.001
HOMA-IR	9.15 ± 4.63	6.55 ± 2.93	-2.60 (-3.96, -1.25)	0.001
SBP (mmHg)	138.2 ± 11.2	132.9 ± 12.3	-5.3 (-7.9, -2.6)	<0.001
DBP (mmHg)	85.9 ± 6.4	83.4 ± 8.0	-2.5 (-4.2, -0.8)	0.005
TG (mmol/L)	2.89 ± 1.74	1.67 ± 0.60	-1.22 (-1.87, -0.57)	0.001
TC (mmol/L)	5.14 ± 1.17	4.40 ± 0.97	-0.75 (-1.27, -0.23)	0.007
LDL-C (mmol/L)	2.90 ± 0.87	2.79 ± 0.78	-0.11 (-0.45, -0.23)	0.511
HDL-C (mmol/L)	1.26 ± 0.23	1.36 ± 0.22	0.10 (0.03, 0.16)	0.006

Normally distributed data expressed as mean ± standard deviation and non-normally distributed data expressed as median or as numbers and percentages. Non-normally distributed data were log-transformed for use with parametric statistics. BMI: body mass index. Relative total body Fat: fat percentage of total body weight. Relative total body Lean: lean percentage of total body weight. SAT: subcutaneous adipose tissue. VAT: visceral adipose tissue. HbA_1c_: glycosylated haemoglobin A_1c_. FBG: fasting blood glucose. P2BG: 2-hour postprandial blood glucose. sFCP: serum fasting C-peptide. CPI: C-peptide index. HOMA-IR: homeostasis model assessment of insulin resistance. CPI = sFCP (ng/mL) × 100/fasting glucose (mg/dL). HOMA-IR = fasting glucose (mg/dL) × fasting insulin (μU/ml)/405. 95% CI: lower 95% confidence interval limit, upper 95% confidence interval limit. SBP: systolic blood pressure. DBP: diastolic blood pressure. TG: triglyceride. TC: total cholesterol. LDL-C: low density lipoprotein-cholesterol. HDL-C: high density lipoprotein-cholesterol.

### DXA assessments

#### Absolute and relative changes in total body fat mass and lean tissue mass

There were significant absolute reductions in both total body fat and lean mass from baseline to post-treatment with liraglutide in the subjects (Table 2). Furthermore, the mean absolute reduction in total fat mass of 3.79 kg was significantly greater than the lean mass of 1.52 kg in the intra-individuals (Table 2). And the relative mean fat mass reduction (percentage of pre-treatment total fat mass) of 12.3% was also greater than the relative lean mass reduction of 2.8% (Table 2). The relative total body fat mass (percentage of total body weight) was reduced by 2.3% from baseline of 37.9% to 35.6%, while the relative total body lean mass was increased by 2.3% from baseline of 62.1% to 64.4%, the changes were significantly different (Table 2). There was no correlation between pre-treatment body weight and the reduction in total body fat tissue (r = -0.052, p = 0.781) and lean tissue (r = 0.02, p = 0.916).

### CT Assessments

#### Visceral and abdominal subcutaneous tissue areas

There were significant reductions in both abdominal VAT and SAT areas from baseline to post-treatment with liraglutide in the subjects (Table 2). The mean absolute reduction in abdominal VAT areas of 42.3 cm^2^ was significantly greater than the SAT areas of 23.3 cm^2^ in the intra-individuals (t = -3.861, p<0.001). And the relative mean reduction in VAT areas (percentage of total abdominal fat mass areas) of 15.2% was also greater than the relative SAT areas reduction of 10.3% (t = -2.605, p = 0.012). In addition, there was a significant change in VAT/SAT ratio before and after treatment (1.41 to 1.34,Table 2). There was no correlation between pre-treatment body weight and the reduction in abdominal VAT areas (r = 0.18, p = 0.333) and SAT areas (r = -0.353, p = 0.052). These data indicated that liraglutide led to weight loss as a result of a reduction in fat tissue, especially in visceral fat tissue.

#### Changes in parameters relating to glucose metabolism following 12-week liraglutide treatment

As expected, 12-week treatment with liraglutide was associated with significant mean reductions in HbA1c of 1.06%, FBG of 1.77 mmol/L and P2BG of 4.39 mmol/L, respectively (Table 2), but it was not seen a significant direct linear correlation between changes in body fat reduction and HbA1c decreased (r = 0.093, p = 0.619). Serum fasting C-peptide and C-peptide index (CPI) were significantly increased in all subjects by liraglutide treatment. Liraglutide also significantly improved insulin resistance indexed by HOMA-IR (Table 2). Furthermore, there was a weak but significant correlation between change in body fat and change in HOMA-IR (r = 0.35, p = 0.042). These data indicated that liraglutide treatment could ameliorate insulin secretion and insulin resistance.

#### Changes in lipid profiles and blood pressure

By the end of the 12-week treatment period, the TG and TC levels were significantly decreased, while the HDL-C level was increased. The mean changes in TG was -1.22 mmol/L (t = -3.926, p = 0.001), TC was -0.75 mmol/L (t = -2.983, p = 0.007) and HDL-C was 0.10 mmol/L (t = 3.075, p = 0.006). However, there was no significant change in the concentration of LDL-C (t = -0.668, p = 0.511). Further more, significant reductions in both the SBP and DBP were observed in present study, the mean changes were -5.3 mmHg (t = -4.008, p<0.001) and -2.5 mmHg (t = -3.053, p = 0.005), respectively (Table 2).

#### Changes in plasma cardiac natriuretic peptides levels

**Figure 1:**
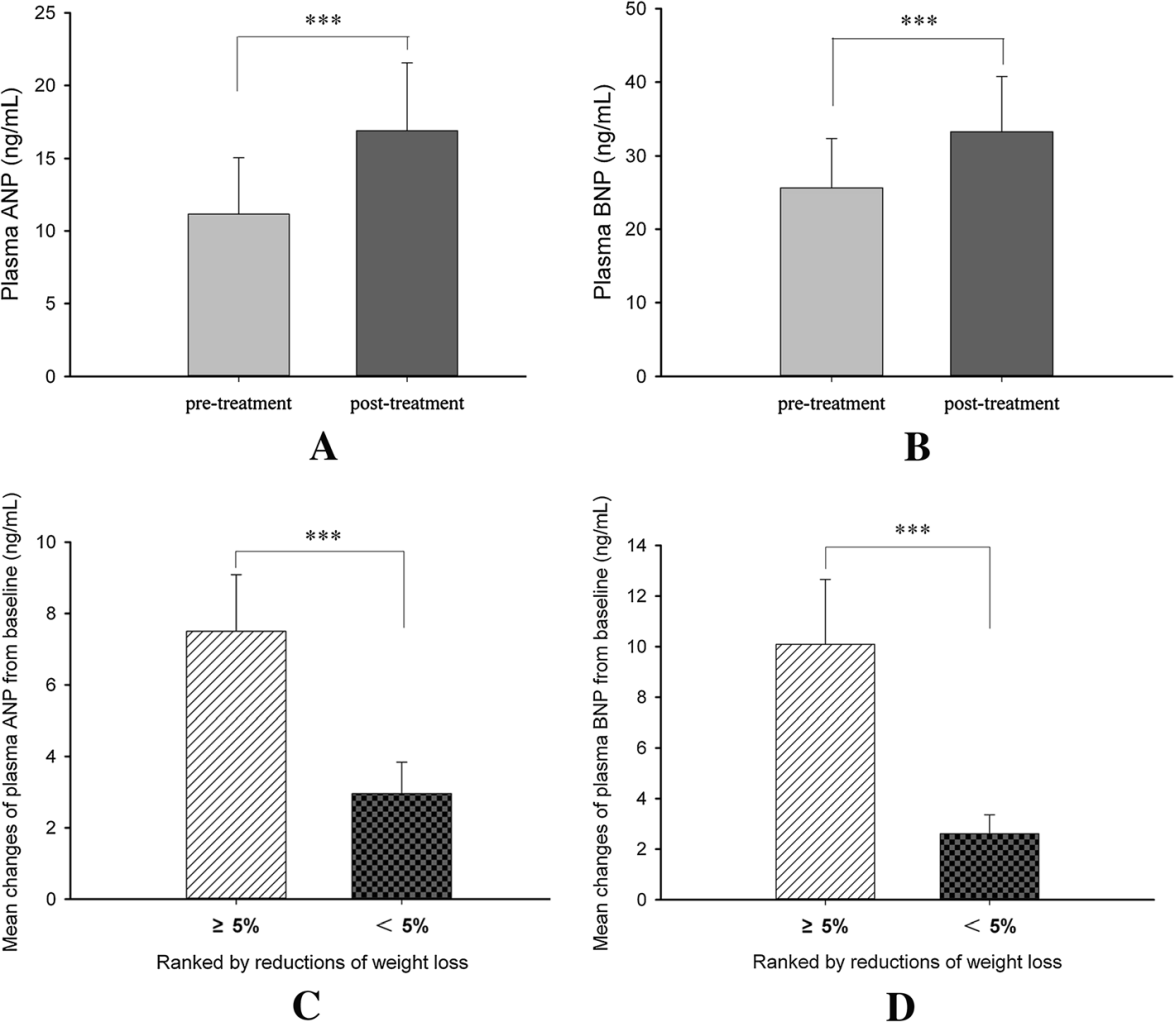
**Changes in the plasma ANP and BNP levels following 12-week liraglutide treatment.** ANP: A-type natriuretic peptides. BNP: B-type ventricular natriuretic peptides. **(A)** and **(B)**: Changes in the plasma ANP and BNP levels at pre-treatment and post-treatment respectively. **(C)** and **(D)**: Comparisons the increases in the ANP and BNP levels within two subgroups stratified by reductions of body weight loss: subjects who lost weight more than 5% and subjects who lost weight less than 5%.

Both the plasma ANP and BNP levels were significantly increased following the 12-week liraglutide treatment (ANP from baseline 11.16 ± 3.89 ng/mL to 16.91 ± 4.64 ng/mL; BNP from baseline 25.64 ± 6.72 ng/mL to 33.29 ± 7.48 ng/mL, respectively, Figure 1A, 1B) in all the subjects. Reinforcing this, when we stratified patients into subgroups according to amount of weight loss < 5% or ≥ 5% [patients with < 5% weight loss (n = 12) and patients with ≥ 5% weight loss (n = 19), the characteristics of the subgroups were seen in Additional file 1: Table S1], there was a significant increase in ANP levels in patients who lost weigh more than 5% compared with those who lost weight less than 5% (7.50 ± 1.59 ng/mL vs. 2.96 ± 0.88 ng/mL, p<0.001, Figure 1C). Similar to ANP levels, the mean increment of BNP levels in the patients who lost weight more than 5% was significant greater than those who lost weight less than 5% (10.09 ± 2.57 ng/mL vs. 2.61 ± 0.75 ng/mL, p<0.001, Figure 1D).

#### Relationships between changes in body compositions and pre-treatment cardiac NPs levels following 12-week liraglutide treatment

Results are presented in Additional file 2: Table S2. Unexpectedly, there was no correlations between pretreatment ANP levels and changes in body compositions following 12-week liraglutide treatment (p = 0.096-0.961). However, BNP levels at pre-treatment were weak but significant related to changes in body weight (r = 0.497, p = 0.004), fat mass (r = 0.487, p = 0.005), relative fat mass (r = 0.497, p = 0.004), lean mass (r = 0.514, p = 0.003) and VAT areas (r = 0.385, p = 0.032), but not related to changes in SAT areas (r = 0.306, p = 0.095).

#### Relationships between changes in body compositions and changes in cardiac NPs levels following 12-week liraglutide treatment

**Figure 2:**
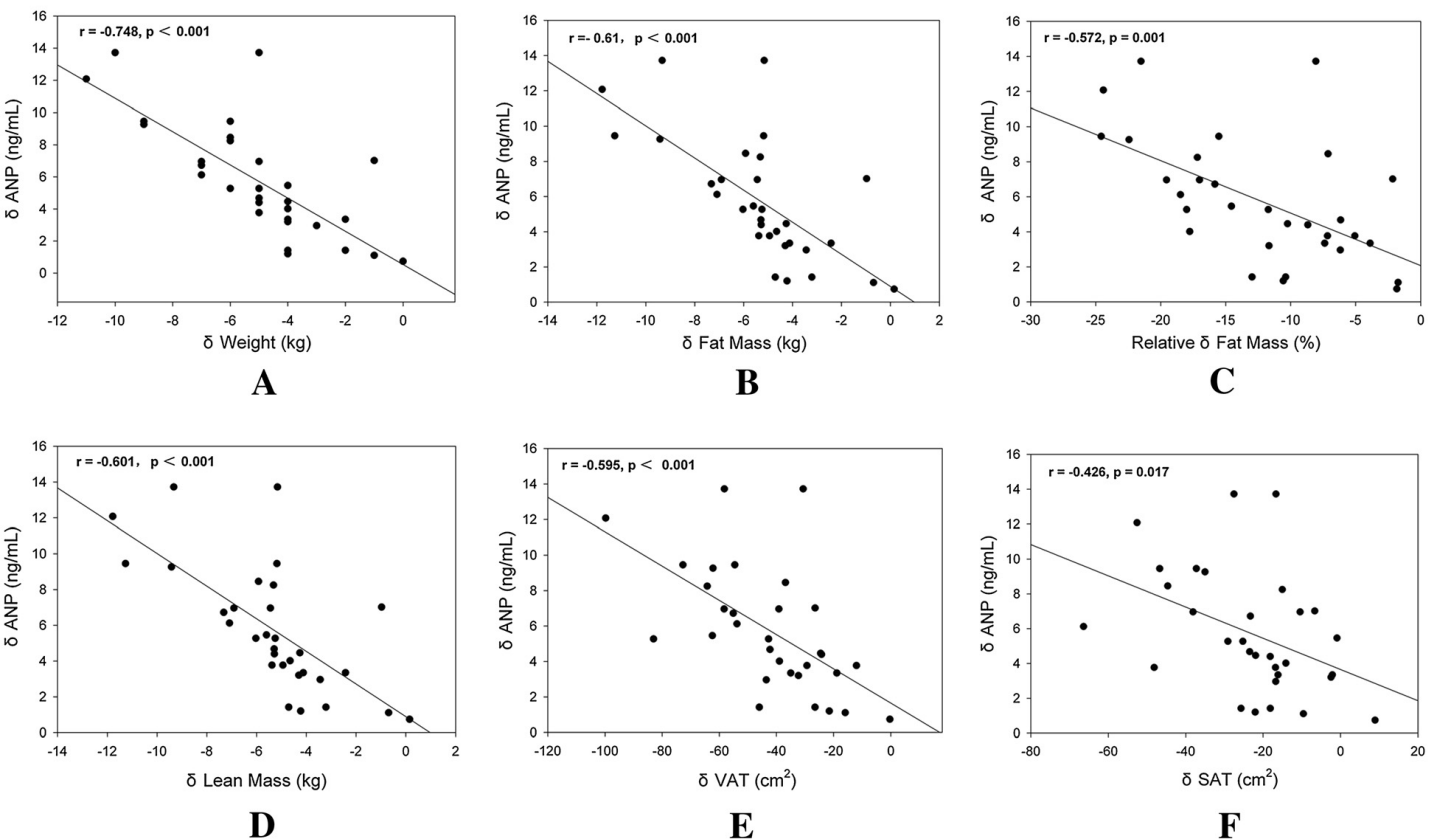
**Correlations between changes in ANP and body compositions following 12-week liraglutide treatment.** Δ: The change of values following liraglutide treatment. ANP: A-type natriuretic peptides. SAT: subcutaneous adipose tissue. VAT: visceral adipose tissue. **(A)**: Change in plasma ANP levels and change in body weight. **(B)**: Change in plasma ANP levels and change in fat mass. **(C)**: Change in plasma ANP levels and change in relative fat mass. **(D)**: Change in plasma ANP levels and change in lean mass. **(E)**: Change in plasma ANP levels and change in VAT areas. **(F)**: Change in plasma ANP levels and change in SAT areas.

**Figure 3:**
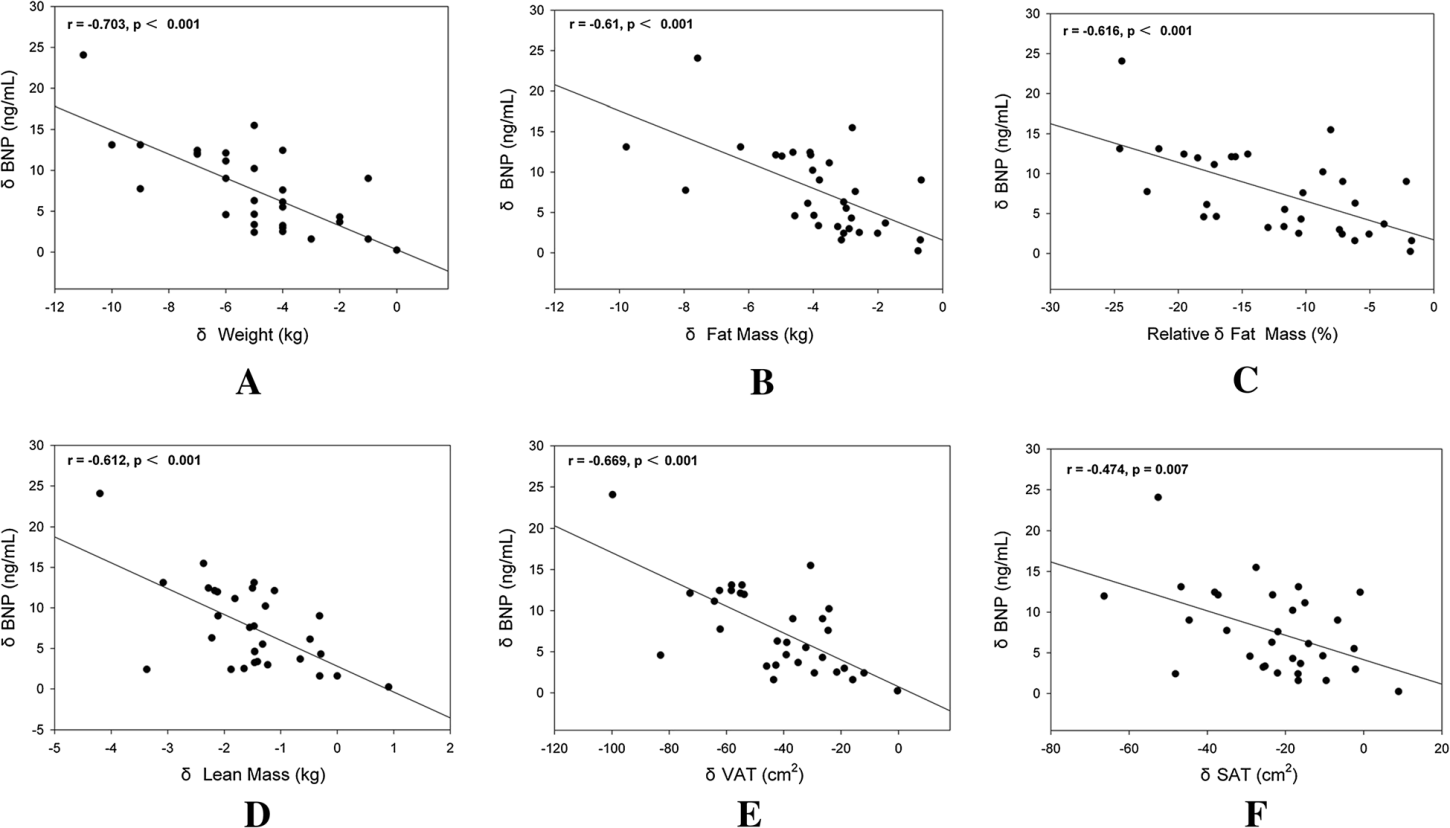
**Correlations between changes in BNP levels and body compositions following 12-week liraglutide treatment.** Δ: The change of values following liraglutide treatment. BNP: B-type natriuretic peptides. SAT: subcutaneous adipose tissue. VAT: visceral adipose tissue. **(A)**: Change in plasma BNP levels and change in body weight. **(B)**: Change in plasma BNP levels and change in fat mass. **(C)**: Change in plasma BNP levels and change in relative fat mass. **(D)**: Change in plasma BNP levels and change in lean mass. **(E)**: Change in plasma BNP levels and change in VAT areas. **(F)**: Change in plasma BNP levels and change in SAT areas.

Results are presented in Additional file 2: Table S2. As hypothesized, there was significant inverse correlations between increases in ANP levels and changes in body weight (r = -0.748, p<0.001, Figure 2A), fat tissue (r = -0.61, p<0.001, Figure 2B), relative fat (r = -0.572, p = 0.001, Figure 2C), lean tissue (r = -0.601, p<0.001, Figure 2D), VAT (r = -0.595, p<0.001, Figure 2E), and SAT (r = -0.426, p = 0.017, Figure 2F). And the significant inverse correlation was also seen in the increase in BNP levels and reductions in total body fat tissue (r = -0.61, p<0.001), relative fat (r = 0.616, p<0.001), lean tissue (r = -0.612, p<0.001), VAT (r = -0.669, p<0.001) and SAT (r = -0.474, p = 0.007) (Figure 3).

## Discussion

In addition to the expected achieving good glycemic control, this study demonstrated that treatment with liraglutide over a 12-week period led to a mean weight loss of 5.03 kg in Chinese obese T2D, only one subject’s weight did not change, and 61.3% of the subjects lost more than 5% of body weight from baseline. We also observed the changes of liraglutide-induced body composition assessed by DXA and CT. It was shown that reductions in body weight with liraglutide primarily came from reductions in fat mass rather than lean mass, and decreases in VAT area seemed greater than in SAT. The most interesting findings reported here were the significant correlations between weight loss and increases in both plasma ANP and BNP levels following 12-week liraglutide treatment. We also found that NPs increased particularly more in patients who lost weight more than 5% compared with patients who lost weight less than 5%, suggesting the possible role of increases in circulation NPs levels on the liraglutide-induced weight loss. Given the “rediscovery” of brown adipocytes in adult humans [24,25] and the potential role of ANP and BNP to stimulate the appearance of brown-like adipocytes associated with increased expenditure, our results imply that increases in plasma NPs may add another dimension to explain how liraglutide induces sustained weight loss.

### Liraglutide-induced weight loss might be associated with increases in plasma NPs concentrations

The LEAD-2 [5] and LEAD-3 [6] studies have shown that liraglutide (as monotherapy or in combination with metformin) resulted in greater reductions in fat mass (1.0–2.4 kg) than in lean mass (1.5 kg), confirming our results in which fat mass decreased 3.8 kg and lean mass decreased 1.5 kg after 12-week liraglutide treatment. In addition, the results from CT assessment were also in line with the results from LEAD-2 [5] shown reductions in VAT area were larger than SAT. These are all desirable traits because increased fat mass are associated with decreased insulin sensitivity and increased morbidity and mortality, furthermore, visceral fat is causally associated with insulin resistance [26]. In this study, we found that there was a significant correlation between change in total body fat and change in HOMA-IR. Noticeable, the reductions in fat mass in our study were greater than those of previous studies in terms of the changes in liraglutide-induced body composition, including the LEAD-2 [5], LEAD-3 [6] studies, as well as a shorter study in which fat mass decreased 1.0 kg after 8-week treatment with liraglutide 0.6 mg once-daily [27]. What are the reasons for the excellent results reported in our study? It might be because body fat distribution differs across ethnic background, Chinese and South Asian cohorts had relatively greater amount of abdominal adipose tissue, and this difference was more pronounced in VAT [28]. It is possible that liraglutide exhibited its utmost effectiveness on weight reduction merely due to genetic differences, confirming the hypothesis of one recent study from Japanese in which liraglutide administrated 0.9 mg once-daily achieved about 10% of body weight reduction after 6 months, the result was also much greater than those mainly done in Caucasians [13,29], however the Japanese study failed to observe the reductions in body fat tissue, and the present study using DXA and CT to assess the changes in liraglutide-induced body compositions only performed in Chinese obese T2D. Therefore, the excellent results of liraglutide-induced reductions in fat tissue should be supported in the future in a double-blind placebo-controlled clinical trial across ethnic groups.

Lowering of energy intake has been shown with liraglutide as well as native GLP-1 both in animals [30,31] and human subjects [10]. In rats, intracerebroventricular administration of GLP-1 has been shown to induce a marked reduction in food intake, presumably by interacting with GLP-1 receptors, which are present in several areas in the central nervous system [32,33]. In pharmacological doses, exogenous administration of GLP-1 has been shown to slow gastric emptying substantially [9]. In clinical studies, it was demonstrated that appetite, food preference and eating behavior were significantly changed by liraglutide treatment [34-36]. Therefore, the underlying mechanisms of liraglutide inducing weight loss are partly related to the combined effects of GLP-1 on the gastrointestinal tract and the brain, leading to delaying gastric emptying, inducing satiety and changing eating behavior to reduce the energy intake.

The effect of liraglutide on energy expenditure is a novel observation that requires further investigation [27,34]. In patients with T2D or not with liraglutide treatment, a weight loss of 2.4 kg ~7.8 kg could be maintained over 52 weeks to 2 years. Since gastrointestinal effects such as vomiting and nauseas were mostly transient, it was apparent that induction of nausea and less energy intake cannot fully explain the liraglutide-induced weight loss in such long term [30]. Recently, it was reported that GLP-1 could increase adipocytes lipolysis in a dose-dependent manner [13]. Moreover, Minsuk Kim et al. have demonstrated that cardiac GLP-1R activation promoted the secretion of atrial natriuretic peptide (ANP) [15]. NPs were also shown to increase lipid oxidation and adipocyte thermogenesis [18], which was subsequently found to promote “browning” of white adipocytes in mouse and human adipocytes through p38 MAPK pathway [37]. In addition, increases in plasma NPs levels were proved to be associated with weight loss induced by gastric bypass surgery [38] and lifestyle changes in human [39], suggesting that increased NPs play an important role in the weight loss. An attractive hypothesis was raised whether the liraglutide-induced weight loss is associated with the increased circulation NPs levels. In our study, we found that increases in both ANP and BNP levels with liraglutide treatment were associated with reductions in body weight. Moreover, the patients who lost weight more than 5% had approximate 2.5-fold and 3.9-fold intra-individual increases in plasma ANP and BNP levels respectively, compared with the patients who lost weight less than 5% (Figure 2C and Figure 2D). Taken together, our data suggested that liraglutide-induced weight loss primarily from reductions in fat mass might be associated with changes in the NP system.

### Possible mechanisms of liraglutide increasing circulating NPs levels

The mechanisms underlying the relative NPs deficiency in obesity are not clear. NP clearance receptors (NPRC) which was found on adipocytes, referred to as the clearance receptor binds ANP and BNP to remove them from circulation, and elevated NPRC have been demonstrated in patients with obesity, suggesting that a putative role for adipose tissue in the clearance of NPs from the circulation [40-42]. This is supported by an animal model of NPRC^-/-^ null mice in which reduced adipose tissue associated with increased NPs has been observed, and the typical brown adipocyte markers were elevated in both BAT and WAT [37]. It is well known that NPs are synthesized and released from the ventricles in response to increased cardiac wall. In this study, we excluded the patients with heart failure at baseline that would account for the observed increase in NPs levels. Moreover, the protective role of GLP-1 in cardiovascular disease has been suggested in both animal experiments and clinical studies. Bao W et al. revealed that the long acting GLP-1 receptor agonist could provid more sustained cadioprotective effect in the setting of acute myocardial I/R injury than the short-acting exendin-4 [43]. Clinically, beneficial effects of GLP-1 have also been demonstrated in patients with heart failure [44]. Endogenous GLP-1 level was found to be increased in patients with high cardiovascular risk, suggesting it represent a contra-regulatory response in states of metabolic disorder, hepertriglyceridemia and insulin resistance [45]. In accordance with those previous studies, we observed that treatment with liraglutide could significantly improve cardiovascular risk factors including triglycride, total cholesterol, HDL cholesterol and blood pressure, but not the LDL cholesterol. A study among the Japanese obese patients with T2D also found a significantly reduction in LDL-C with liraglutide at 6 months after discharge [35]. In addition, a recent study demonstrated that liraglutide led to a reduction of blood pressure through activating the secretion of ANP, but this effect was eliminated in the GLP-1R deficient mouse model, suggesting that liraglutide regulate blood pressure via GLP-1R and ANP axis dependent way [15]. We therefore hypothesized that the increases in the NPs levels were not secondary to underlying cardiac changes, but attributed to the liraglutide treatment. With a deeper molecular and clinical understanding of how liraglutide increase NPs levels to regulate fat metabolism and cardiovascular function in the future, the researchers may find new ways to manage obese T2D.

### Limitations and conclusion

Several limitations of the present study deserve mention. The study involves a relative small sample size and an observational design without use of control subjects. The future study need to confirm our results, which should be to power a larger, more definitive study incorporating appropriate controls. This might include a patient group treated with an agent that improves glycemic control but not induce weight loss and a second group in whom weight loss alone is achieved (e.g. orlistat) to determine the independent effect on increases in NPs levels following liraglutide treatment. Additionally, the future study should be designed to observe the longitudinal changes of NPs following liraglutide-induced weight loss.

In summary, we found there was a significant correlation between the increases in NPs levels and the reductions in weight loss following the 12-week liraglutide treatment. We also showed that NPs increased particularly more so in patients who lost weight more than 5% compared with patients who lost weight less than 5%. Our data imply that increases in plasma NPs may add another novel dimension to explain how liraglutide induces sustained weight loss. It would seem that a much better understanding of the molecular basis for changes in NPs following the weight loss induced by liraglutide is needed in the future.

## Abbreviations

ANP: Atrial natriuretic peptides; BAT: Brown adipose tissue; BMI: Body mass index; BNP: B-type ventricular natriuretic peptides; CPI: C-peptide index; CT: Computerized tomography; CVD: Cardiovascular disease; DBP: Diastolic blood pressure; DPP-IV: Dipeptidyl peptidase-IV; DXA: Dual-energy X-ray absorptiometry; ELISA: Enzyme linked immuno sorbent assay; FBG: Fasting blood glucose; GLP-1: Glucagon-like peptide-1; HDL-C: High density lipoprotein-cholesterol; HOMA-IR: Homeostasis model assessment of insulin resistance; LDL-C: Low density lipoprotein-cholesterol; LEAD: Liraglutide Effect and Action in Diabetes; NPRC: NP clearance receptors; NPs: Natriuretic peptides; P2BG: 2-hour postprandial blood glucose; SAT: Subcutaneous adipose tissue; SBP: Systolic blood pressure; sFCP: Serum fasting C-peptide; T2D: Type 2 diabetes; TC: Total cholesterol; TG: Triglyceride; TZDs: Thiazolidinediones; VAT: Visceral adipose tissue; WAT: White adipose tissue

## Competing interest

The authors declare that they have no competing interest.

## Authors’ contribution

DMY, QY and SL conceived the study, analyzed data and wrote the manuscript. CJL and PY acquired and analyzed data, and wrote the manuscript. CJL and QMZ acquired and researched data. All authors read and approved the final manuscript.

## Supplementary Material

Additional file 1: Table S1 Characteristics of subgroup subjects. Click here for file

Additional file 2: Table S2 Correlations between changes in body compositions and plasma NPs levels following 12-week liraglutide treatment. Click here for file
