# Supplementary material for: Changes in liraglutide-induced body composition are related to modifications in plasma cardiac natriuretic peptides levels in obese type 2 diabetic patients
Source: Cardiovasc Diabetol. 2014 Feb 5;13:36. doi: 10.1186/1475-2840-13-36 (PMC3923001; doi:10.1186/1475-2840-13-36)
Supplement: Additional file 1: Table S1 — Characteristics of subgroup subjects. [file 1475-2840-13-36-S1.doc]

Supplementary table 1 characteristics of subgroup subjects

|  | weight loss ＜ 5% (n=12) | |  | weight loss ≥ 5% (n=19) | |
| --- | --- | --- | --- | --- | --- |
|  | Pre-treatment | Post- treatment |  | Pre-treatment | Post-treatment |
| Sex (M/F) | 5/7 | 5/7 |  | 11/8 | 11/8 |
| Age (years) | 45.8±12.9 |  |  | 50.3±10.4 |  |
| Duration of diabetes (years) | 7.5±3.9 |  |  | 6.5±4.3 |  |
| FBG (mmol/L) | 8.09±1.39 | 6.83±0.61 |  | 8.97±0.95 | 6.88±0.82 |
| P2BG (mmol/L) | 11.86±2.65 | 8.90±1.15 |  | 13.80±2.54 | 8.51±0.71 |
| HbA_1c_ (%) | 7.98±0.73 | 7.09±0.49 |  | 8.33±0.79 | 7.15±0.59 |
| Body weight (kg) | 96.0±12.5 | 93.1±11.9 |  | 88.8±11.7 | 82.4±11.4* |
| BMI (kg/m^2^) | 33.1±3.8 | 32.07±3.7 |  | 30.8±3.3 | 28.6±3.2* |
| Waist circumference (cm) | 112.2±8.9 | 110.3±9.8 |  | 106.6±8.2 | 102.9±7.9* |
| Abdominal VAT area（cm2） | 279.6±68.3 | 252.2±80.6 |  | 276.7±70.9 | 225.0±64.7 |
| Abdominal SAT area（cm2） | 252.2±80.6 | 235.4±80.3 |  | 187.6±76.5# | 160.2±73.1* |
| VAT:SAT ratio | 1.19±0.42 | 1.16±0.44 |  | 1.56±0.70# | 1.48±0.74* |
| Total fat mass (kg) | 38.4±11.8 | 36.1±11.8 |  | 30.9±8.4 | 26.1±8.2* |
| Total lean mass (kg) | 54.8±9.0 | 53.9±8.8 |  | 54.9±8.3 | 53.0±8.2 |
| ANP levels (ng/mL) | 11.75±4.75 | 14.70±4.53 |  | 10.79±3.32 | 18.30±4.25* |
| BNP levels (ng/mL) | 26.92±7.61 | 30.71±7.36 |  | 24.83±6.17 | 34.92±7.27 |

Normally distributed data expressed as mean ± standard deviation and non-normally distributed data expressed as median or as numbers and percentages. Non-normally distributed data were log-transformed for use with parametric statistics. HbA_1c_: glycosylated haemoglobin A_1c_. FBG: fasting blood glucose. P2BG: 2-hour postprandial blood glucose. BMI: body mass index. Relative total body Fat: fat percentage of total body weight. Relative total body Lean: lean percentage of total body weight. SAT: subcutaneous adipose tissue. VAT: visceral adipose tissue. ANP: A-type natriuretic peptides. BNP: B-type ventricular natriuretic peptides. #p＜ 0.05 compared the difference between the subgroup at baseline , *p ＜ 0.05 compared the changes between the subgroup at post-treatment
