# Supplementary material for: Changes in liraglutide-induced body composition are related to modifications in plasma cardiac natriuretic peptides levels in obese type 2 diabetic patients
Source: Cardiovasc Diabetol. 2014 Feb 5;13:36. doi: 10.1186/1475-2840-13-36 (PMC3923001; doi:10.1186/1475-2840-13-36)
Supplement: Additional file 2: Table S2 — Correlations between changes in body compositions and plasma NPs levels following 12-week liraglutide treatment. [file 1475-2840-13-36-S2.doc]

**Supplementary table 2 Correlations between changes in body compositions and plasma NPs levels following 12-week** liraglutide treatment

|  | Pre-treatment ANP levels | |  | Pre-treatment BNP levels | |  | Δ ANP levels | |  | Δ BNP levels | |
| --- | --- | --- | --- | --- | --- | --- | --- | --- | --- | --- | --- |
|  | *r* value | *p* value |  | *r* value | *p* value |  | *r* value | *p* value |  | *r* value | *p* value |
| Δ body weight | 0.135 | 0.47 |  | 0.497 | 0.004 |  | -0.748 | 0.000 |  | -0.703 | 0.000 |
| Δ fat tissue | 0.03 | 0.871 |  | 0.487 | 0.005 |  | -0.61 | 0.000 |  | -0.61 | 0.000 |
| Δ relative fat | 0.036 | 0.849 |  | 0.497 | 0.004 |  | -0.572 | 0.001 |  | -0.616 | 0.000 |
| Δ lean tissue | 0.305 | 0.096 |  | 0.514 | 0.003 |  | -0.601 | 0.000 |  | -0.612 | 0.000 |
| Δ VAT | 0.009 | 0.961 |  | 0.385 | 0.032 |  | -0.595 | 0.000 |  | -0.669 | 0.000 |
| Δ SAT | 0.249 | 0.177 |  | 0.306 | 0.095 |  | -0.426 | 0.017 |  | -0.474 | 0.000 |

Δ: The change of values following liraglutide treatment; ANP: atrial natriuretic peptides ; BNP: ventricular natriuretic peptides ; SAT: subcutaneous adipose tissue; VAT: visceral adipose tissue.
